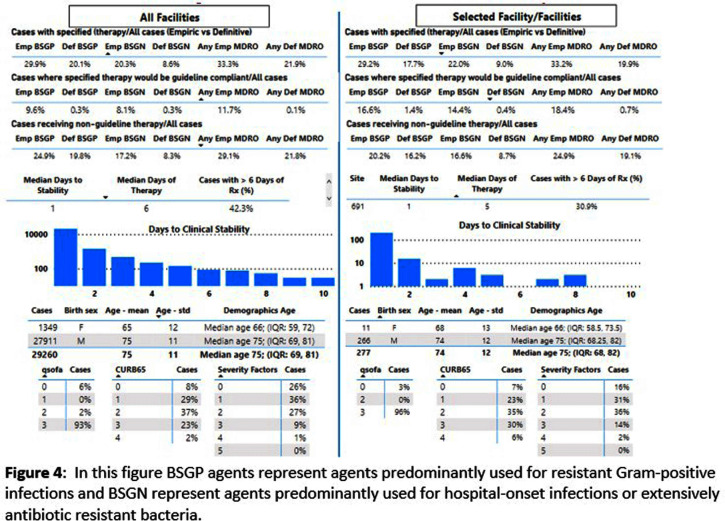# Guideline-unjustified inpatient therapy for non-ICU patients with community-acquired pneumonia (CAP) at 105 Veterans Affairs

**DOI:** 10.1017/ash.2024.151

**Published:** 2024-09-16

**Authors:** Matthew Goetz, Christopher Graber, Melinda Neuhauser, Makoto Jones

**Affiliations:** UCLA; VA Greater Los Angeles Healthcare System; CDC DHQP

## Abstract

**Background:** CAP is often inappropriately treated with agents active against multidrug-resistant organisms (MDRO; methicillin-resistant S. aureus [MRSA] and P. aeruginosa [PSA]) and for prolonged duration. We assessed the relationship between antibiotic use with ATS/IDSA guideline-unjustified empiric and definitive MDRO therapy and prolonged duration in non-ICU inpatients with CAP at 105 VA Medical Centers. **Methods:** From VA Corporate Data Warehouse data, we identified patients with discharge ICD-10-CM codes consistent with CAP from 1/2022-3/2023, excluding cases with 14 days of antibiotic therapy, ICU admission, concurrent infections, or severe immunocompromise. We considered as jultified empiric (≤third day of hospitalization) therapy: anti-MRSA therapy for patients with prior positive MRSA cultures, anti-PSA therapy for patients with prior positive PSA cultures, and both anti-MRSA & anti-PSA therapy in patients with severe pneumonia and intravenous antibiotics in the prior 3 months. Definitive (>third day of hospitalization) anti-MDRO therapy was considered unjustified in patients who had achieved clinical stability and whose cultures did not grow MRSA or PSA. Prolonged duration (>6 days of therapy) was unjustified if patients were clinically stable or discharged by day 5. **Results:** The median age of the 29,260 patients was 75 (IQR 69,81); 4.6% were women. While 33% and 22% of patients received empiric or definitive MDRO therapy, such therapy was jultified in 12% and 0.5%, respectively. Median facility use of empiric and definitive MDRO therapy was 31% (IQR 25%,38%) and 20% (15%,23%), respectively (Figure 1); this use was unjustified in 89% (85%,93%) and 100% (100%,100%), respectively. Pearson’s correlation coefficient between MDRO therapy and rates of unjustified empiric and definitive MDRO therapy for CAP was 0.54 and 0.61, respectively (Figure 2). Although 99% of patients were discharged or stable by day 5, 42% received prolonged therapy. The median frequency of prolonged therapy was 39% (33%,48%); facility rates of prolonged therapy had a correlation of 0.56 with total antibiotic use and 0.46 with MDRO therapy (Figure 3). **Discussion:** Based on electronic documentation, we identified 1) substantial opportunities to reduce unjustified anti-MDRO therapy and the duration of therapy in hospitalized non-ICU patients with CAP; 2) a moderate correlation of unjustified anti-MDRO therapy with increased MDRO antibiotic use and of prolonged duration of therapy with increased total and MDRO antibiotic use. The correlation of lower quality prescribing with increased antibiotic use provides further impetus for tools such as dashboards (Figure 4) to assist antibiotic stewards in designing and monitoring interventions to reduce unjustified therapy.

**Figure:**
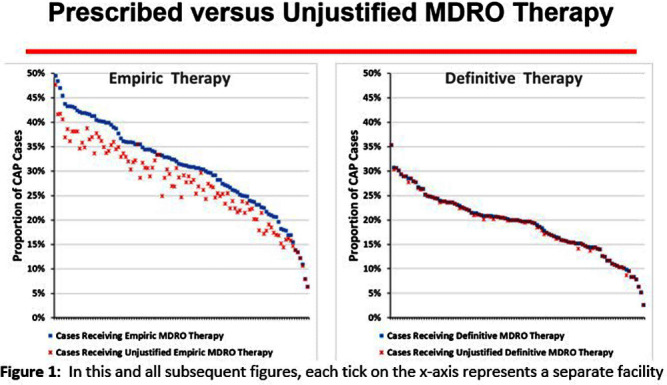

**Figure:**
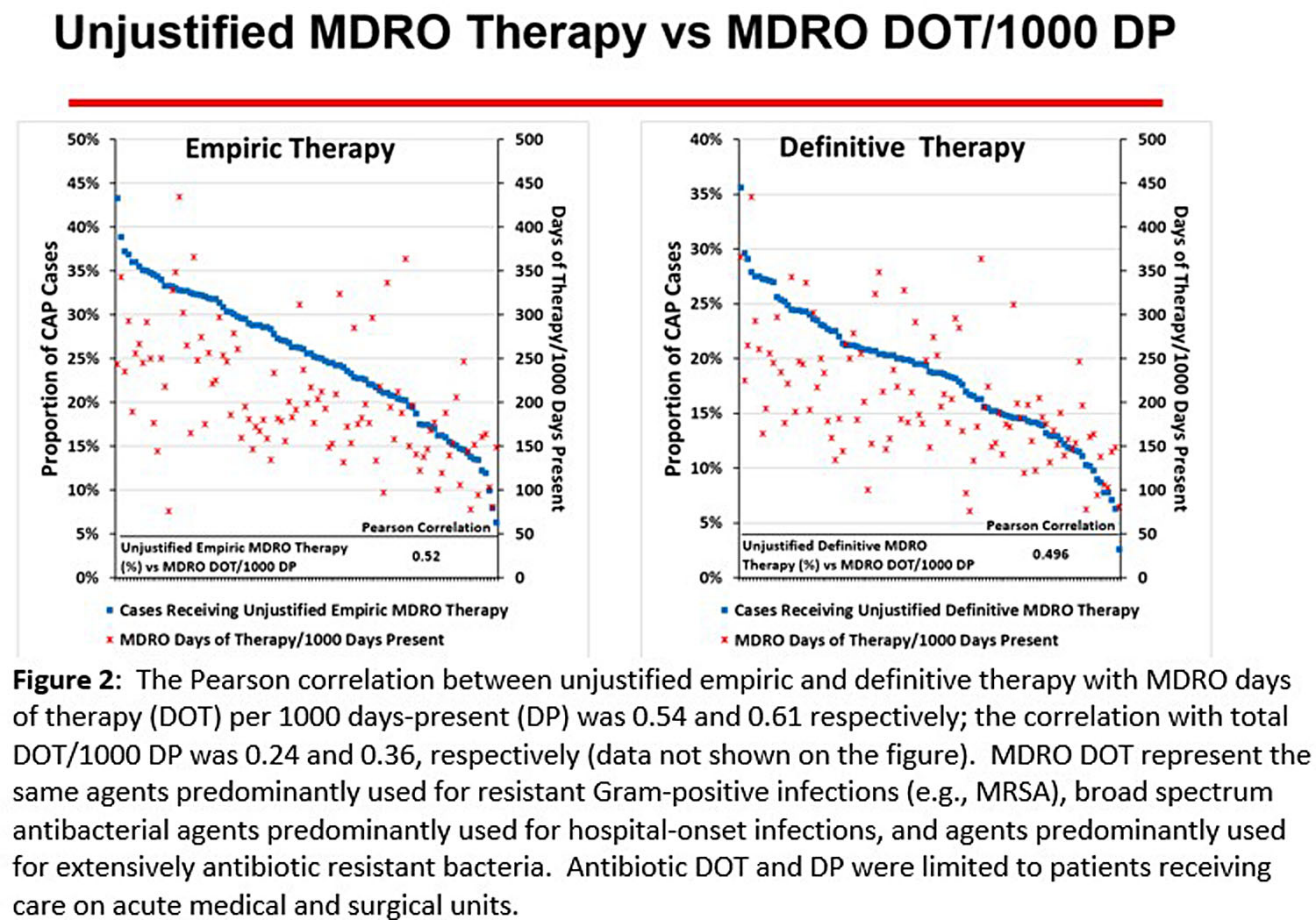

**Figure:**
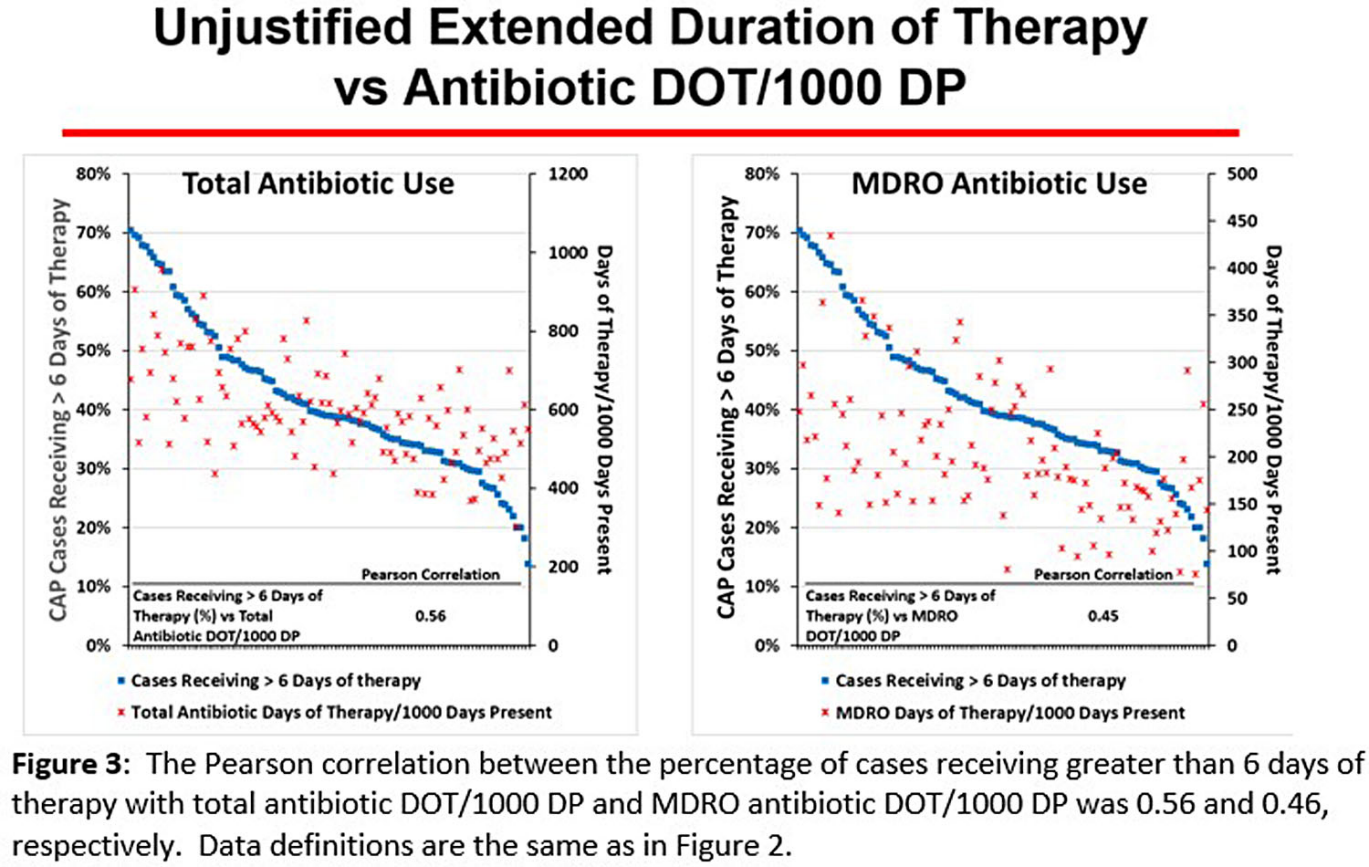

**Figure:**